# PF-05280014 (a trastuzumab biosimilar) plus paclitaxel compared with reference trastuzumab plus paclitaxel for HER2-positive metastatic breast cancer: a randomised, double-blind study

**DOI:** 10.1038/s41416-018-0340-2

**Published:** 2018-12-20

**Authors:** Mark D. Pegram, Igor Bondarenko, Marina Moreira Costa Zorzetto, Sachin Hingmire, Hirotaka Iwase, Petr V. Krivorotko, Keun Seok Lee, Rubi K. Li, Joanna Pikiel, Rajesh Aggarwal, Reginald Ewesuedo, Amy Freyman, Ray Li, Alicia Vana, Donghua Yin, Charles Zacharchuk, Elizabeth Tan-Chiu

**Affiliations:** 1 Stanford Comprehensive Cancer Institute, Stanford University School of Medicine, 900 Blake Wilbur, Stanford, CA 94305 USA; 2 Oncology and Medical Radiology Department, Dnipropetrovsk Medical Academy, 31 Blyzhnya Street, Dnipro, 49102 Ukraine; 3 Department of Clinical Oncology, Barretos Cancer Hospital, Rua Antenor Duarte Villela, 1331 – Dr. Paulo Prata, Barretos, São Paulo 14784-400 Brazil; 4 Department of Oncology, Deenanath Mangeshkar Hospital and Research Centre, Erandawne, Pune, Maharashtra 411004 India; 5 Department of Breast and Endocrine Surgery, Kumamoto University Graduate School of Medical Science, 1-1-1, Honjo, Chuo-ku Kumamoto, 860-8556 Japan; 6 Department of Breast Tumors, Petrov Research Institute of Oncology, 68 Leningradskaya Street, Pesochny 197758 Saint Petersburg, Russian Federation; 7 Center for Breast Cancer, National Cancer Center, 323 Ilsan-ro, Ilsandong-gu, Goyang-si, Gyeonggi-do Republic of Korea; 8 Section of Medical Oncology, Cancer Institute, St. Luke’s Medical Center, 279 E Rodriguez Sr. Ave, Quezon City, 1112 Metro Manila Philippines; 9 Copernicus, Wojewódzkie Centrum Onkologii, Al. Zwycięstwa 31/32, 80-210, Gdańsk, Poland; 10 Pfizer Essential Health, Worldwide Safety, Pfizer Inc, 235 East 42nd Street, New York, NY 10017 USA; 11 Pfizer Essential Health, Biosimilars Clinical R&D, Pfizer Inc, 610 Main Street, Cambridge, MA 02139 USA; 12 Early Clinical Development Statistics, Pfizer Inc, 1 Portland Street, Cambridge, MA 02139 USA; 13 Pfizer Essential Health, Biosimilars Clinical R&D, Pfizer Inc, 10777 Science Center Drive, San Diego, CA 92121 USA; 14 Clinical Pharmacology, Pfizer Inc, 10777 Science Center Drive, San Diego, CA 92121 USA; 15 Medical Oncology, Florida Cancer Research Institute, 201 NW 82nd Avenue, Suite 102, Plantation, FL 33324 USA

**Keywords:** Randomized controlled trials, Breast cancer, Breast cancer, Targeted therapies, Antibody therapy

## Abstract

**Background:**

This randomised, double-blind study compared PF-05280014 (a trastuzumab biosimilar) with reference trastuzumab (Herceptin®) sourced from the European Union (trastuzumab-EU), when each was given with paclitaxel as first-line treatment for HER2-positive metastatic breast cancer.

**Methods:**

Between 4 April 2014 and 22 January 2016, 707 participants were randomised 1:1 to receive intravenous PF-05280014 plus paclitaxel (PF-05280014 group; *n* = 352) or trastuzumab-EU plus paclitaxel (trastuzumab-EU group; *n* = 355). PF-05280014 or trastuzumab-EU was administered weekly (first dose 4 mg/kg, subsequent doses 2 mg/kg), with the option to change to a 3-weekly regimen (6 mg/kg) from Week 33. Treatment with PF-05280014 or trastuzumab-EU could continue until disease progression. Paclitaxel (starting dose 80 mg/m^2^) was administered on Days 1, 8 and 15 of 28-day cycles for at least six cycles or until maximal benefit of response. The primary endpoint was objective response rate (ORR), evaluating responses achieved by Week 25 and confirmed by Week 33, based on blinded central radiology review.

**Results:**

The risk ratio for ORR was 0.940 (95% CI: 0.842–1.049). The 95% CI fell within the pre-specified equivalence margin of 0.80–1.25. ORR was 62.5% (95% CI: 57.2–67.6%) in the PF-05280014 group and 66.5% (95% CI: 61.3–71.4%) in the trastuzumab-EU group. As of data cut-off on 11 January 2017 (using data up to 378 days post-randomisation), there were no notable differences between groups in progression-free survival (median: 12.16 months in the PF-05280014 group vs. 12.06 months in the trastuzumab-EU group; 1-year rate: 54% vs. 51%) or overall survival (median: not reached in either group; 1-year rate: 89.31% vs. 87.36%). Safety outcomes and immunogenicity were similar between the treatment groups.

**Conclusion:**

When given as first-line treatment for HER2-positive metastatic breast cancer, PF-05280014 plus paclitaxel demonstrated equivalence to trastuzumab-EU plus paclitaxel in terms of ORR.

**CLINICAL TRIAL REGISTRATION:**

ClinicalTrials.gov, NCT01989676

## Background

Trastuzumab is a recombinant, humanised, monoclonal antibody that targets a juxta-membrane epitope in subdomain IV of the human epidermal growth factor receptor 2 (HER2) extracellular domain (ECD). Trastuzumab has been shown in both in vitro and in vivo assays to inhibit the proliferation of HER2-overexpressing human tumour cells, and it is a potent mediator of antibody-dependent cell-mediated cytotoxicity.^1,2^ Clinically, trastuzumab is used in the treatment of HER2-overexpressing breast and gastric cancers.^1,2^ Compared with chemotherapy alone, the addition of trastuzumab to chemotherapy has been associated with survival benefits in both early-stage and metastatic breast cancer (MBC),^3,4^ as well as gastric cancer.^5^ However, physicians have reported barriers to patients accessing trastuzumab, which may result in suboptimal treatment.^6^

Biosimilars—biological products that are highly similar to a licensed reference (i.e., originator) biologic—provide additional treatment options that may expand access to biologics such as trastuzumab.^6^ To gain regulatory approval, a potential biosimilar must be shown to have no clinically meaningful differences in quality attributes, efficacy or safety compared with the originator product.^7–9^ Biosimilars are developed in a stepwise process that involves head-to-head comparison with the originator, and biosimilarity is determined based on the totality of evidence from analytical, nonclinical and clinical studies.^7–9^

The humanised monoclonal antibody PF-05280014 is a biosimilar to the trastuzumab reference product Herceptin®. In the European Union (EU), PF-05280014 has been authorised under the name Trazimera™ (Pfizer Europe MA EEIG, Sandwich, UK) for the same indications as reference trastuzumab.^2,10^ PF-05280014 has an identical amino acid sequence to reference trastuzumab, and has demonstrated similarity in comparative structural, functional and nonclinical in vivo studies.^11^ Furthermore, a single-dose comparative study in healthy male volunteers established similarity in pharmacokinetics (PK),^12^ and a comparative trial in the neoadjuvant HER2-positive (HER2+) breast cancer setting evaluated PK, efficacy, safety and immunogenicity.^13^

As the final step in the biosimilarity assessment, we conducted the current study (REFLECTIONS B327–02) to compare the efficacy, safety and immunogenicity of PF-05280014 and reference trastuzumab (Herceptin®) sourced from the EU (trastuzumab-EU), each in combination with paclitaxel, as first-line treatment for patients with HER2+ MBC. The primary objective was to compare objective response rate (ORR) in the two treatment groups, testing the hypothesis that the risk ratio for ORR was within a pre-specified equivalence margin. Here, we report results of the primary efficacy analysis, conducted after all patients had completed Week 33 tumour assessments (or discontinued study treatment earlier). We also present secondary analyses based on a later data cut-off date, when all patients had completed Week 53 tumour assessments (or discontinued study treatment earlier).

## Methods

### Study design and participants

This is an ongoing, international, randomised, double-blind, parallel-group study (ClinicalTrials.gov: NCT01989676; EudraCT: 2013-001352-34). Patients were randomised at 143 sites across 24 countries (Argentina, Brazil, Chile, Czech Republic, Greece, Hungary, India, Japan, Latvia, Mexico, Peru, Philippines, Poland, Portugal, Republic of Korea, Romania, Russian Federation, Serbia, Slovakia, South Africa, Thailand, Turkey, Ukraine, and the United States).

Eligible patients were females aged ≥ 18 years with metastatic, histologically confirmed breast cancer with at least one measurable lesion as defined by Response Evaluation Criteria in Solid Tumours (RECIST) 1.1. Patients were required to have documented HER2+ status according to one of the following: HER2 gene amplification by fluorescent *in-situ* hybridisation (FISH), chromogenic *in-situ* hybridisation (CISH) or dual *in-situ* hybridisation (DISH), with amplification defined per the manufacturer’s kit instruction; HER2 overexpression by immunohistochemistry (IHC) categorised as IHC3+; or HER2 overexpression by IHC categorised as IHC2+, with FISH, CISH or DISH confirmation. For study entry, HER2+ status could be established via local or central laboratory assessment; however, all patients were required to have available tumour tissue for central review of HER2 status (see Supplementary Methods S1 for additional details on determination of HER2 positivity). Patients had documented oestrogen receptor (ER) status (positive or negative) based on local or central laboratory assessment. Additionally, patients were required to have an Eastern Cooperative Oncology Group performance status of 0–2, screening laboratory values within specified limits, and left ventricular ejection fraction (LVEF) within the institution’s normal range, by echocardiogram or multi-gated acquisition scan.

Exclusion criteria included prior systemic therapy for MBC (except endocrine therapy); relapse within 1 year of last dose of previous adjuvant or neoadjuvant treatment (except endocrine therapy); prior cumulative dose of doxorubicin >400 mg/m^2^ or epirubicin >800 mg/m^2^; inflammatory breast cancer; superficial disease site that could not be assessed radiographically as the only site of measurable disease; major surgery, radiotherapy or any investigational agents within 4 weeks prior to the first dose of study treatment; concurrent administration of other anti-cancer therapies (other than bisphosphonates or anti-RANK [receptor activator of nuclear factor kappa B] ligand antibody to control pre-existing bone metastases); active uncontrolled or symptomatic central nervous system metastases; and active uncontrolled cardiac disease.

Based on a retrospective review of patients randomised during the early part of the study, a requirement for sites to forward radiographs to the independent central review laboratory to confirm the presence of measurable disease prior to patient randomisation was added in a protocol amendment. A full listing of eligibility criteria is provided in Supplementary Methods S2.

### Randomisation and blinding

Using an automated interactive web-based response system, investigators or their pre-specified designee randomised patients in a 1:1 ratio to PF-05280014 plus paclitaxel or trastuzumab-EU plus paclitaxel. Randomisation was stratified by prior trastuzumab exposure and ER status, with a block size of four. This was according to a randomisation schedule that was computer-generated by the sponsor, to which the sponsor’s personnel directly involved in study conduct were blinded. Patients, investigators, central radiology reviewers and the sponsor’s study team were blinded to treatment assignments, and external packaging for each vial of PF-05280014 or trastuzumab-EU appeared identical. Safety data were reviewed in an unblinded manner by an external data monitoring committee throughout the study (up to the Week 53 milestone). Safety outcomes were also reviewed by the sponsor’s study team, who were blinded until database release for the Week 53 analysis.

### Treatments

**Fig. 1:**
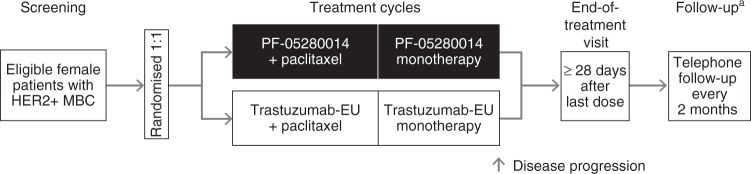
Study design. PF-05280014 or trastuzumab-EU: administered weekly (4 mg/kg loading dose on Cycle 1 Day 1; subsequent doses 2 mg/kg) on Days 1, 8, 15 and 22 of each 28-day cycle during the paclitaxel administration period and until at least Week 33. Following completion of the paclitaxel administration period and beginning no earlier than Week 33, the PF-05280014 or trastuzumab-EU regimen could be changed to 6 mg/kg every 3 weeks. Treatment with PF-05280014 or trastuzumab-EU could continue until disease progression. Paclitaxel: administered on Days 1, 8 and 15 of each 28-day cycle (starting dose 80 mg/m^2^, with provision for dose reduction). In the absence of disease progression or unacceptable toxicity in the judgement of the investigator, paclitaxel treatment was continued for ≥ 6 cycles or until maximal benefit of response was obtained. On days when both treatments were administered, the order of administration was PF-05280014 or trastuzumab-EU infusion followed by paclitaxel infusion. ^a^Follow-up to assess survival status continued until death or until 1 year from randomisation and ≥ 6 months following last dose of study treatment. *HER2+* human epidermal growth factor receptor 2-positive, *MBC* metastatic breast cancer, *trastuzumab-EU* reference trastuzumab sourced from the European Union

PF-05280014 or trastuzumab-EU was administered weekly (first dose 4 mg/kg, intravenous [IV] infusion over 90 min; subsequent doses 2 mg/kg, infused over 30–90 min, depending on tolerability) on Days 1, 8, 15 and 22 of each 28-day cycle when given in combination with paclitaxel, and until at least Week 33 (Fig. 1). The dose and weekly schedule used in the current trial demonstrated efficacy in a pivotal phase III study of reference trastuzumab in MBC,^3^ and is consistent with the labelling for reference trastuzumab in Europe and the United States.^1,2^ Following completion of the paclitaxel administration period, and beginning no earlier than Week 33, the PF-05280014 or trastuzumab-EU regimen could be changed at the investigator’s discretion to 6 mg/kg, infused over 30–90 min every 3 weeks. This approach ensured consistent treatment during the evaluation period for the primary efficacy endpoint while allowing for a less-frequent treatment schedule after this time. Treatment could continue until disease progression, as assessed by RECIST 1.1, in the judgement of the investigator. Dose reductions of PF-05280014 and trastuzumab-EU were not permitted.

Paclitaxel was administered on Days 1, 8 and 15 of each 28-day cycle (starting dose 80 mg/m^2^, IV infusion over 60 min). All patients were to receive premedication for paclitaxel administration in accordance with local paclitaxel product information and standard of care. In the absence of disease progression or unacceptable toxicity in the judgement of the investigator, paclitaxel treatment was continued for at least six cycles or until maximal benefit of response was obtained. Depending on toxicity, provision was made for paclitaxel dose reductions.

### Outcomes and assessments

Per regulatory guidance, as a scientific matter, ORR was considered sufficiently sensitive to detect any clinically meaningful differences in efficacy between PF-05280014 and trastuzumab-EU in this study population. Thus, the primary endpoint for the study was ORR, defined as the percentage of patients in each group with complete or partial response by Week 25 that was confirmed by Week 33, in accordance with RECIST 1.1. Blinded central radiology review was the basis for the primary analysis. Secondary efficacy endpoints included duration of response (DOR; the time from first documentation of objective tumour response to progressive disease or death), 1-year progression-free survival (PFS) rate (based on the time from randomisation to progressive disease or death) and 1-year overall survival rate (based on the time from randomisation to death). PFS and DOR evaluations were also based on blinded central radiology assessments. Additional secondary endpoints to support the assessment of similarity were safety, peak and trough concentrations of PF-05280014 and trastuzumab-EU at selected cycles and incidence of antidrug antibodies (ADAs), including neutralising antibodies (NAbs).

Computed tomography or magnetic resonance imaging scans of the chest and abdomen, and any other site of disease clinically indicated, were required at screening. During treatment, tumour assessments were performed every 8 weeks to Week 41, at Week 53 and then every 12 weeks (or sooner if clinically indicated). The allowable window for tumour imaging assessments was ± 14 days. All scans through Week 53 were forwarded for central radiology review. Patients with tumour response were required to undergo confirmatory assessments ≥ 4 weeks later. Bone scans were carried out at screening for all patients, and then repeated during the treatment phase if clinically indicated or to confirm response if lesions were identified at screening.

Serum samples for determining peak and trough drug concentrations were collected at Cycle 1, 3, 4, 5, 7, 8 and subsequently every 3 cycles until the end-of-treatment visit (28 days post last study treatment administration). Samples were collected pre-dose at each scheduled cycle (Week 1 Day 1). Additional samples were collected during Cycles 1 and 5, 1 h after the end of infusion (Week 1 Day 1) and pre-dose at Week 2 Day 8. Samples were analysed by QPS LLC (Newark, DE, USA) using a validated, sensitive and specific enzyme-linked immunosorbent assay (ELISA). Sample concentrations were determined by interpolation from calibration standard curves in the range 0.500–100 μg/ml. The concentration–time data were used for a population PK analysis using a nonlinear mixed effect modelling approach (to be reported separately).

For assessment of ADAs and NAbs, serum samples were collected pre-dose at Cycle 1, 3, 5, 8 and subsequently every 3 cycles until the end-of-treatment visit. Two electrochemiluminescent immunoassays, one for detecting antibodies against PF-05280014 and the other for detecting antibodies against trastuzumab-EU, were used to analyse the samples. Both ADA assays utilised the same immunoassay platform and were validated in accordance with draft US Food and Drug Administration guidance on assay development and validation for immunogenicity testing of therapeutic protein products.^14^ ADA analyses were conducted by Intertek Pharmaceutical Services (San Diego, CA, USA), and followed a tiered approach of screening, confirmation and titre determination.^12^ If a sample tested positive for ADA according to the dosed product, the sample was also tested for cross-reactivity of the ADA against the other product, using the corresponding assay. Samples testing positive for the presence of ADAs (anti-PF-05280014 or anti-trastuzumab-EU) were further analysed for NAbs. NAb analyses were conducted by QPS LLC (Newark, DE, USA), using validated competitive binding assays.

Additionally, serum samples were collected pre-dose at Cycle 1, 3, 5, 8 and at the end-of-treatment visit, and analysed by Q^2^ Solutions (Morrisville, NC, USA) for soluble, shed HER2 ECD concentrations using a HER2 ELISA development kit (Nuclea Diagnostics, Pittsfield, MA, USA).

Safety was characterised by the type, incidence, severity, timing, seriousness and relationship to study treatment of adverse events (AEs) and laboratory abnormalities. Additional evaluations included vital signs, 12-lead electrocardiograms and LVEF assessment by echocardiogram or multi-gated acquisition scan. Investigators recorded AEs, their severity (graded in accordance with the National Cancer Institute Common Terminology Criteria for Adverse Events version 4.03^15^) and the investigator’s opinion of their relationship to study treatment. Medical Dictionary for Regulatory Activities v19.1 coding was applied. Signs and symptoms of infusion-related reactions (IRRs) were defined as such by the investigator on the AE reporting form, in addition to the investigator recording a separate AE of IRR. AEs of special interest were identified based on the established safety profile of reference trastuzumab,^1,2^ with the listing finalised prior to the data cut-off for the primary efficacy analysis. Treatment-emergent AEs (TEAEs) were those that occurred (or worsened, if pre-existing) after the beginning of study treatment through 70 days after the last dose. Serious AEs (SAEs) were those that resulted in death, were life-threatening, required hospitalisation (or prolongation of existing hospitalisation) or resulted in disability, incapacity or congenital abnormality.

After treatment discontinuation, patient survival status was collected by telephone every 2 months until death or until 1 year from randomisation and ≥ 6 months following last dose of study treatment.

### Statistical analyses

The primary efficacy (Week 33) analysis was prospectively planned to be performed after all randomised patients had received treatment to Week 25 and undergone assessment for confirmation of response by Week 33, or discontinued study treatment before Week 33. Similarity between PF-05280014 and trastuzumab-EU was demonstrated if the 95% confidence interval (CI) of the risk ratio for ORR was within a pre-specified equivalence margin of 0.80–1.25, as per the method proposed by Miettinen and Nurminen.^16^ The equivalence margin was derived from a publication-level meta-analysis (unpublished) of three randomised studies of reference trastuzumab used in combination with taxanes.^3,17,18^ Using a random effect model, the overall estimated log-transformed risk ratio of ORR of chemotherapy alone over trastuzumab plus chemotherapy was −0.54, with a one-sided 90% upper confidence bound of −0.32. A 75% fraction of the upper bound was taken, resulting in a log-transformed risk ratio of −0.24. This value was exponentiated to a risk ratio of 0.79, which corresponded to a margin of 0.79–1.27 for equivalence testing; the more conservative range of 0.80–1.25 was used. Assuming an ORR of 60% in both treatment groups (a conservative estimate based on a separate, unpublished, publication-level meta-analysis), a sample of 630 patients (*n* *=* 315/arm) provided ~85% power to demonstrate equivalence with a 2.5% type 1 error rate. Allowing for 10% attrition, a total of 690 randomised patients (*n* = 345/arm) was planned. With an estimated ORR of 60% in the trastuzumab-EU group and a sample of 630 patients, the chosen margin would result in an ORR acceptance range of 55.0–66.4% in the PF-05280014 group for claiming equivalence. This acceptance range for ORR was considered to be clinically acceptable for determining similarity in the efficacy of PF-05280014 and trastuzumab-EU.

For the ORR calculation, if a patient had a missing tumour outcome that prevented the derivation of best overall response, the patient was considered a non-responder. The primary analysis was repeated with the additional stratification variables to assess whether prior trastuzumab exposure or ER status would affect the risk ratio; response rates based on investigator assessments were also calculated. Additionally, in a post-hoc analysis, the risk difference for ORR was calculated, along with its 95% CI.

The secondary (Week 53) analyses presented were based on clinical data collected up to 378 days post-randomisation, as of a cut-off date when all patients had either completed Week 53 tumour assessments or discontinued study treatment earlier. For secondary efficacy endpoints, the Kaplan–Meier method was used to estimate PFS rate, overall survival rate and DOR, and a log-rank test stratified by prior trastuzumab exposure and ER status was used to compare the treatments. Hazard ratios (HRs) and 95% CIs based on a Cox proportional hazard model with stratification by prior trastuzumab exposure and ER status were presented. For time-to-event endpoints, missing data were censored. Safety data were summarised using descriptive statistics, as were serum drug and HER2 ECD concentration–time data. The percent change from baseline in serum HER2 ECD concentrations was also calculated. The percentage of patients with positive ADA and NAb results was summarised for each treatment group.

The intent-to-treat (ITT) population, defined as all patients randomised to study treatment, was used for the efficacy analyses. The per protocol (PP) population was defined as all patients with no major protocol deviations who had HER2+ MBC as confirmed by central review, measurable disease at baseline as confirmed by central review, and who were randomised and received study treatment as planned per protocol. The PP population was used for sensitivity analyses of efficacy outcomes (using the statistical methods outlined above) and for evaluation of serum soluble HER2 ECD levels. The safety population, defined as all patients who received at least one dose of study treatment, was used for safety and immunogenicity analyses. The PK population was defined as all patients treated with PF-05280014 or trastuzumab-EU who had no major protocol deviations that influenced PK assessments and had at least one postdose concentration measurement.

No interim analysis was planned. As the study is ongoing, the data presented are based on two pre-planned analyses conducted for the Week 33 and Week 53 milestones while the study database remains open; database lock will take place when long-term follow-up is complete.

A statement on the availability of study data can be found in the “Additional Information” section.

## Results

### Patient disposition and baseline characteristics

**Fig. 2:**
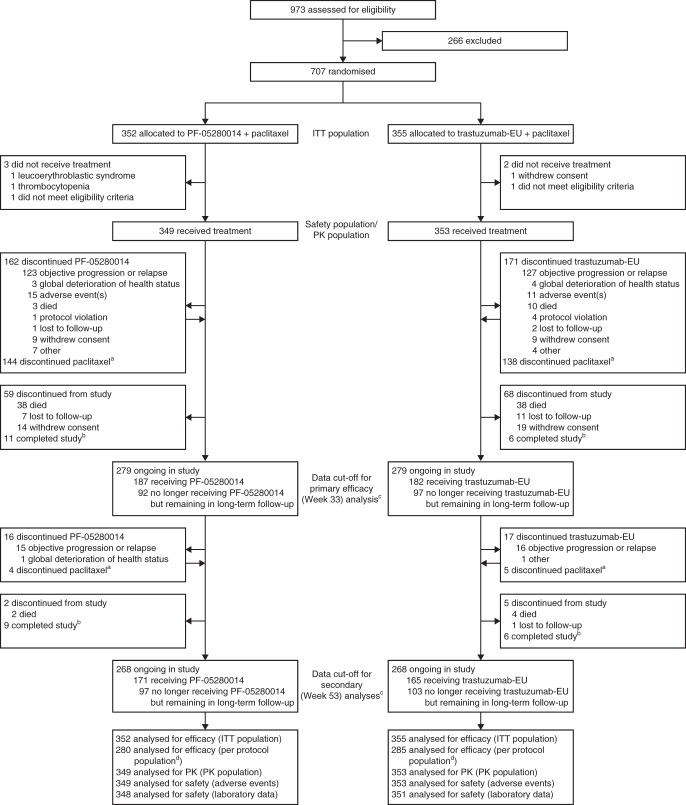
Study profile. Primary efficacy analysis based on a data cut-off date (24 August 2016) when all patients had either completed Week 33 tumour assessments or discontinued study treatment earlier. Secondary analyses based on a data cut-off date (11 January 2017) when all patients had either completed Week 53 tumour assessments or discontinued study treatment earlier. ^a^Number does not include patients who completed treatment with paclitaxel per protocol. ^b^Patients who completed all follow-up as required by the protocol. ^c^Status at time of data cut-off, using data up to 378 days post-randomisation. ^d^Reasons for exclusion from the per protocol population were comparable between the groups. The most frequent reason for exclusion was “no measurable disease at baseline per central reviewer” (31 [8.8%] patients in the PF-05280014 group vs. 25 [7.0%] patients in the trastuzumab-EU group), followed by “missing, not evaluable or equivocal HER2 status by central laboratory” (27 [7.7%] vs. 25 [7.0%] patients). *HER2* human epidermal growth factor receptor 2, *ITT* intent-to-treat, *PK* pharmacokinetics, *trastuzumab-EU* reference trastuzumab sourced from the European Union

Of the 973 patients screened, 707 patients were randomised to PF-05280014 plus paclitaxel (PF-05280014 group; *n* = 352) or trastuzumab-EU plus paclitaxel (trastuzumab-EU group; *n* = 355) between 4 April 2014 and 22 January 2016, and comprised the ITT population. In total, 439 (62.1%) of the 707 enrolled patients were randomised per the protocol amendment that required sites to obtain central confirmation of measurable disease before treatment allocation (222 [63.1%] patients in the PF-05280014 group and 217 [61.1%] patients in the trastuzumab-EU group). Five patients were randomised but did not receive study treatment (three in the PF-05280014 group and two in the trastuzumab-EU group). Figure 2 summarises the populations analysed and patient dispositions, which were similar between the treatment groups. Baseline characteristics and demographics were well-balanced (Table 1).

**Table 1:** Baseline demographic and other characteristics (ITT population)

	PF-05280014 plus paclitaxel (*n* = 352)	Trastuzumab-EU plus paclitaxel (*n* = 355)	Total (*N* = 707)
Age, years			
Mean (SD)	54.0 (10.8)	54.1 (10.9)	54.1 (10.8)
Median (range)	55.0 (19–80)	54.0 (25–85)	54.0 (19–85)
Weight, kg			
Mean (SD)	69.1 (17.1)	68.1 (16.1)	68.6 (16.6)
Median (range)	68.2 (29–147)	66.0 (36–139)	67.0 (29–147)
Race, *n* (%)			
White	232 (65.9)	244 (68.7)	476 (67.3)
Black	5 (1.4)	8 (2.3)	13 (1.8)
Asian	104 (29.5)	84 (23.7)	188 (26.6)
Other	11 (3.1)	19 (5.4)	30 (4.2)
Time since initial diagnosis of breast cancer^a^			
Mean (SD), months	24.8 (37.81)	22.4 (29.83)	23.6 (34.02)
Missing, *n*	9	7	16
Histopathological classification, *n* (%)			
Ductal	278 (79.0)	277 (78.0)	555 (78.5)
Lobular	14 (4.0)	17 (4.8)	31 (4.4)
Unknown	4 (1.1)	3 ( < 1.0)	7 ( < 1.0)
Other	56 (15.9)	58 (16.3)	114 (16.1)
Disease site,^b^ *n* (%)			
Lung	186 (52.8)	185 (52.1)	371 (52.5)
Liver	146 (41.5)	166 (46.8)	312 (44.1)
Lymph node	259 (73.6)	252 (71.0)	511 (72.3)
Skin	45 (12.8)	33 (9.3)	78 (11.0)
Bone	183 (52.0)	177 (49.9)	360 (50.9)
Brain	4 (1.1)	4 (1.1)	8 (1.1)
Breast	192 (54.5)	191 (53.8)	383 (54.2)
Other	68 (19.3)	82 (23.1)	150 (21.2)
Oestrogen receptor status, *n* (%)			
Positive	184 (52.3)	184 (51.8)	368 (52.1)
Negative	168 (47.7)	171 (48.2)	339 (47.9)
Prior trastuzumab exposure, *n* (%)			
Yes	33 (9.4)	39 (11.0)	72 (10.2)
No	319 (90.6)	316 (89.0)	635 (89.8)
ECOG score, *n* (%)			
0	186 (52.8)	194 (54.6)	380 (53.7)
1	150 (42.6)	146 (41.1)	296 (41.9)
2	16 (4.5)	15 (4.2)	31 (4.4)
LVEF result, %			
Mean (SD)	65.4 (5.84)	65.3 (6.20)	65.3 (6.02)

*ECOG* Eastern Cooperative Oncology Group, *ITT* intent-to-treat, *LVEF* left ventricular ejection fraction, *SD* standard deviation, *trastuzumab-EU* reference trastuzumab sourced from the European Union

^a^Defined as time from initial diagnosis to first dose on Cycle 1 Day 1

^b^Data for disease sites recorded as “No” or “Not Assessed” are not presented

The data cut-off date for the primary efficacy analysis was 24 August 2016. Of the 702 patients who received study treatment, 538 (76.6%) were exposed to at least eight cycles of treatment with PF-05280014 (266 [76.2%] patients) or trastuzumab-EU (272 [77.1%] patients). The data cut-off date for the secondary (Week 53) analyses presented was 11 January 2017. At that time, 536 (75.8%) of the 707 randomised patients remained ongoing in the study: 336 (47.5%) were still being actively treated with PF-05280014 or trastuzumab-EU, and 200 (28.3%) had discontinued treatment but were being followed for survival. The primary reason for discontinuing treatment with PF-05280014 or trastuzumab-EU was objective disease progression (138 [39.2%] vs. 143 [40.3%] patients, respectively).

### Efficacy

In the primary efficacy analysis, the risk ratio for ORR by Week 25 (confirmed by Week 33) in the ITT population was 0.940 (PF-05280014 group over trastuzumab-EU group), with a 95% CI of 0.842–1.049. Hence, the pre-specified definition of similarity between PF-05280014 and trastuzumab-EU was met. ORR was 62.5% (95% CI: 57.2–67.6%) in the PF-05280014 group and 66.5% (95% CI: 61.3–71.4%) in the trastuzumab-EU group (Table 2). The risk difference for ORR (PF-05280014 group minus trastuzumab-EU group) was −4.0% (95% CI: −11.0% to 3.1%). Sensitivity analyses using stratification factors, the PP population and investigator-rated assessments all supported results of the primary analysis (Supplementary Tables S1–S3).

**Table 2:** Analysis of objective response rate derived from central radiology assessments (ITT population) – Week 33 analysis

	PF-05280014 plus paclitaxel (*n* = 352)	Trastuzumab-EU plus paclitaxel (*n* = 355)	Risk ratio estimate (95% CI)^a^
Objective response rate^b^			
*n* (%)	220 (62.5)	236 (66.5)	0.940
(95% CI)	(57.2–67.6)	(61.3–71.4)	(0.842–1.049)
Best overall response category,^c^ *n* (%)			
Complete response	10 (2.8)	13 (3.7)	
Partial response	210 (59.7)	223 (62.8)	
Stable disease	76 (21.6)	74 (20.8)	
Progressive disease	18 (5.1)	11 (3.1)	
Indeterminate	38 (10.8)	34 (9.6)	

*CI* confidence interval, *ITT* intent-to-treat, *RECIST* Response Evaluation Criteria in Solid Tumours, *trastuzumab-EU* reference trastuzumab sourced from the European Union

^a^Risk ratio and associated 95% CI were based on the Miettinen and Nurminen^16^ method

^b^Defined as the percentage of patients within each treatment group who achieved complete response or partial response by Week 25 that was subsequently confirmed by Week 33 (or early discontinuation), in accordance with RECIST 1.1

^c^Best overall response was determined using data up to and including Week 33

**Fig. 3:**
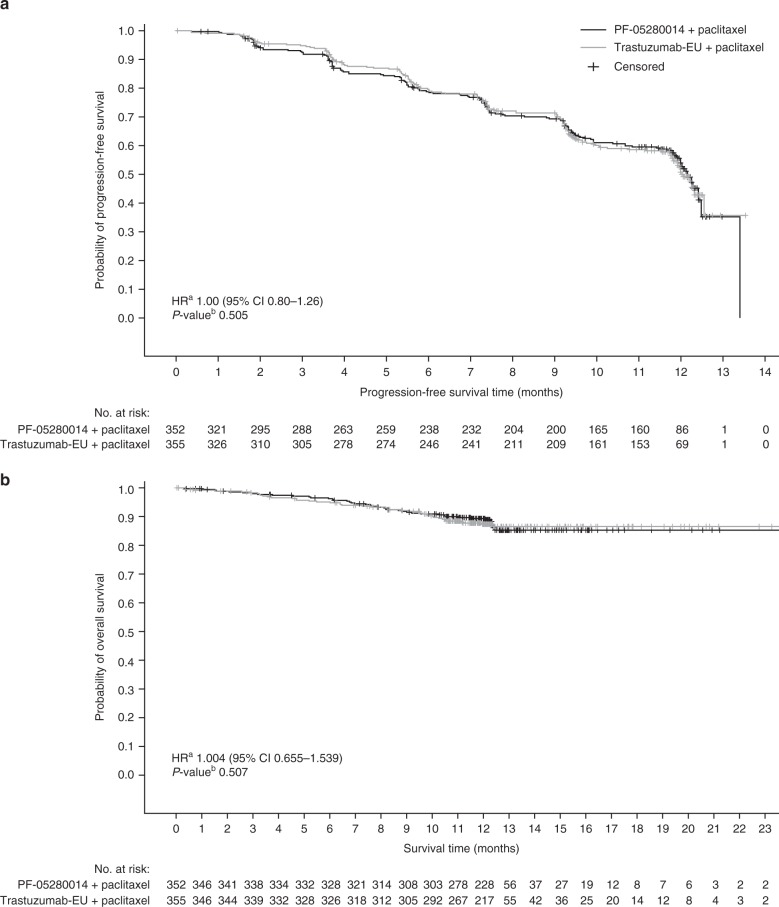
Kaplan–Meier plots of (**a**) progression-free survival (based on central radiology assessments) and (**b**) overall survival (ITT population) – Week 53 analysis. ^a^HRs from a Cox proportional hazards model with prior trastuzumab exposure and oestrogen receptor status as strata. Assuming proportional hazards, an HR < 1 indicates an increased hazard rate for trastuzumab-EU plus paclitaxel; an HR > 1 indicates an increased hazard rate for PF-05280014 plus paclitaxel. ^b^One-sided *P* value from stratified log-rank test. *CI* confidence interval, *HR* hazard ratio, *ITT* intent-to-treat, *trastuzumab-EU* reference trastuzumab sourced from the European Union

In the Week 53 analysis, disease progression or death in the ITT population was reported for 144 (40.9%) patients in the PF-05280014 group and 148 (41.7%) patients in the trastuzumab-EU group. The corresponding estimated 1-year PFS rates were 54% (95% CI: 48–60%) and 51% (95% CI: 45–57%), and median times to PFS were 12.16 (95% CI: 11.93–12.48) months and 12.06 (95% CI: 11.79–not estimable) months (Fig. 3a). The HR for PFS was 1.00 (95% CI: 0.80–1.26), and the stratified log-rank test resulted in a one-sided *P*-value of 0.505, indicating no statistically significant difference between treatments. Estimated 1-year overall survival rates were 89.31% (95% CI: 85.48–92.17%) in the PF-05280014 group and 87.36% (95% CI: 83.27–90.51%) in the trastuzumab-EU group; it was not possible to estimate the median time to death, owing to too few events (Fig. 3b). The HR for overall survival was 1.004 (95% CI: 0.655–1.539; log-rank *P* = 0.507). Median DOR was 11.27 (95% CI: 10.41–11.27) months in the PF-05280014 group and 10.58 (95% CI: 10.22–not estimable) months in the trastuzumab-EU group. The HR for DOR was 0.92 (95% CI: 0.67–1.27; log-rank *P* = 0.304). Sensitivity analyses of secondary efficacy endpoints in the PP population yielded results consistent with those in the ITT population (Supplementary Table S4).

### Safety

In the Week 53 analysis, the majority of patients in the safety population experienced one or more TEAE (337 [96.6%] patients in the PF-05280014 group and 339 [96.0%] patients in the trastuzumab-EU group) (Table 3). The incidence for all categories of TEAEs and serious TEAEs was similar across treatment groups. The most frequently reported TEAEs (≥ 20% of patients in either group) were alopecia, anaemia, neutropenia and peripheral sensory neuropathy. In total, there were 249 (34.5%) patients with a TEAE reported at Grade 3 or higher, with a similar incidence across treatment groups. In the PF-05280014 group, there were 86 (24.6%), 18 (5.2%) and 16 (4.6%) patients with maximum Grade 3, Grade 4 and Grade 5 TEAEs, respectively, and in the trastuzumab-EU group, the corresponding numbers were 96 (27.2%), 9 (2.5%) and 24 (6.8%). The most frequently reported TEAE of Grade 3 or higher in both the PF-05280014 and trastuzumab-EU groups was neutropenia (35 [10.0%] vs. 28 [7.9%] patients).

**Table 3:** Summary of treatment-emergent adverse events (safety population) – Week 53 analysis

	PF-05280014 plus paclitaxel (*n* = 349)	Trastuzumab-EU plus paclitaxel (*n* = 353)	Total (*N* = 702)
Number of TEAEs^a^	2336	2436	4772
Patients with event, *n* (%)			
Any TEAEs	337 (96.6)	339 (96.0)	676 (96.3)
Grade 3 or higher TEAEs	120 (34.4)	129 (36.5)	249 (35.5)
Serious TEAEs^b^	53 (15.2)	56 (15.9)	109 (15.5)
Trastuzumab-related TEAEs	104 (29.8)	101 (28.6)	205 (29.2)
Trastuzumab-related Grade 3 or higher TEAEs	9 (2.6)	11 (3.1)	20 (2.8)
Trastuzumab-related serious TEAEs	5 (1.4)	5 (1.4)	10 (1.4)
TEAEs resulting in permanent discontinuation of trastuzumab	16 (4.6)	12 (3.4)	28 (4.0)
TEAEs with incidence ≥ 10% in either treatment group,^c^ *n* (%)			
Alopecia	189 (54.2)	185 (52.4)	374 (53.3)
Anaemia	120 (34.4)	131 (37.1)	251 (35.8)
Neutropenia	99 (28.4)	91 (25.8)	190 (27.1)
Peripheral sensory neuropathy	93 (26.6)	83 (23.5)	176 (25.1)
Diarrhoea	56 (16.0)	66 (18.7)	122 (17.4)
Nausea	53 (15.2)	64 (18.1)	117 (16.7)
Asthenia	50 (14.3)	43 (12.2)	93 (13.2)
Fatigue	44 (12.6)	49 (13.9)	93 (13.2)
Headache	41 (11.7)	52 (14.7)	93 (13.2)
Leukopenia	36 (10.3)	41 (11.6)	77 (11.0)
Arthralgia	41 (11.7)	36 (10.2)	77 (11.0)
ALT increased	33 (9.5)	41 (11.6)	74 (10.5)
Ejection fraction decreased	35 (10.0)	39 (11.0)	74 (10.5)
Upper respiratory tract infection	30 (8.6)	40 (11.3)	70 (10.0)
Oedema peripheral	24 (6.9)	43 (12.2)	67 (9.5)

“Trastuzumab” refers to PF-05280014 or trastuzumab-EU.

*ALT* alanine aminotransferase, *MedDRA* Medical Dictionary for Regulatory Activities, *TEAE* treatment-emergent adverse event, *trastuzumab-EU* reference trastuzumab sourced from the European Union

^a^TEAE was defined as any event that occurred on or after the first dose of study treatment administration or any pre-existing event that worsened in severity after dosing. TEAE was defined through last dose of trastuzumab + 70 days. For number of TEAEs, the event of “infusion-related reaction” was counted; however, the number of associated signs and symptoms of infusion-related reactions was counted separately (data not presented)

^b^Serious TEAE determined by investigator’s assessment of serious

^c^TEAEs presented in descending order of frequency based on incidence in the total safety population. Patients are only counted once per treatment for each row. MedDRA (v19.1) coding dictionary applied

Overall, 70 (20.1%) patients in the PF-05280014 group and 73 (20.7%) patients in the trastuzumab-EU group experienced at least one SAE. The three most frequently reported SAEs were disease progression (32 [9.2%] patients in the PF-05280014 group vs. 27 [7.6%] patients in the trastuzumab-EU group), pulmonary embolism (5 [1.4%] vs. 3 [0.8%] patients) and pneumonia (4 [1.1%] vs. 3 [0.8%] patients). Of the 85 deaths reported overall (42 [12.0%] vs. 43 [12.2%] patients), 70 were due to MBC (38 [10.9%] vs. 32 [9.1%] patients).

There were no notable differences between treatment groups in the incidences of AEs of special interest, including IRRs, cardiac failure and decreased ejection fraction. The TEAE of IRR was reported for 34 (9.7%) patients in the PF-05280014 group and 30 (8.5%) patients in the trastuzumab-EU group. Signs and symptoms of IRRs were collected in addition to the recorded AE of IRR for those events considered related to PF-05280014 or trastuzumab-EU, respectively, and were reported for 31 (8.9%) and 27 (7.6%) patients. The most frequently reported signs and symptoms were chills (13 [3.7%] vs. 13 [3.7%] patients) and pyrexia (13 [3.7%] vs. 8 [2.3%] patients).

Decreased ejection fraction was reported for 35 (10.0%) patients in the PF-05280014 group and 39 (11.0%) patients in the trastuzumab-EU group; four (1.1%) and five (1.4%) patients had events of Grade 3 or higher, respectively. Mean LVEF values were generally comparable across treatment groups (Supplementary Table S5). The TEAE of cardiac failure occurred in five (1.4%) patients in the PF-05280014 group and seven (2.0%) patients in the trastuzumab-EU group. With the exception of two patients with a Grade 3 event (one of which persisted; the outcome of the other was unknown), the events for the remaining three patients in the PF-05280014 group were Grade 1 or 2. Two patients in the trastuzumab-EU group had a Grade 3 event of cardiac failure (one of which persisted; the other resolved), and two patients had Grade 5 cardiac failure (one with preceding Grade 4 cardiac failure); the events for the remaining three patients were Grade 1. In addition to these TEAEs of cardiac failure, one patient reported acute cardiac failure (Grade 5) and one patient reported congestive heart failure (Grade 2; persistent); both patients were in the trastuzumab-EU group.

No clinically significant differences in laboratory values or electrocardiogram results were observed between the two treatment groups.

### PK, biomarker and immunogenicity analyses

In the Week 53 analysis, mean trough and peak serum concentrations in the PK population were similar for both treatments at all respective time points from baseline up to Cycle 5 Day 8 (Supplementary Figure S1). Per protocol, patients could be on different regimens of PF-05280014 or trastuzumab-EU (either weekly or 3-weekly administration) as of Cycle 9 Day 1, but 12 patients were incorrectly switched as early as Cycle 7 Day 1; hence, concentration comparisons later than Cycle 5 could be confounded by the different regimens and are not presented.

The percent change from baseline in serum soluble HER2 ECD concentrations in the PP population was comparable between the two treatment groups at all subsequent time points through Cycle 8 (Supplementary Table S6).

In the safety population, 30 (8.6%) patients in the PF-05280014 group and 14 (4.0%) patients in the trastuzumab-EU group tested positive for ADAs prior to initiation of treatment. Of these, 20 (5.7%) and nine (2.6%) patients, respectively, tested positive for NAbs. Of the 44 ADA-positive patients at baseline, three patients in the PF-05280014 group and one patient in the trastuzumab-EU group reported prior trastuzumab exposure. All patients tested negative for ADAs after study treatment initiation through 378 days post-randomisation, except two patients (one from each group). Both these patients tested positive for ADAs and NAbs at the end-of-treatment visit; no events of IRR or anaphylactic reaction were reported. One of these two patients was also ADA-positive and NAb-positive at baseline. Drug concentrations for both patients were below the lower limit of quantification at the end-of-treatment visit when ADAs were detected. Prior to the end-of-treatment visit, serum drug concentrations for both ADA-positive patients were within the observed concentration range at corresponding time points for the respective treatment group, although drug concentrations for the ADA-positive patient in the trastuzumab-EU group appeared to be at the lower end of the range for the treatment group.

## Discussion

This study demonstrated similarity between PF-05280014 and trastuzumab-EU in terms of ORR, when each drug was combined with paclitaxel in the first-line treatment of HER2+ MBC. The 95% CI of the risk ratio for ORR was contained within the pre-specified equivalence margin.

In terms of methodological strengths, the study used a randomised, double-blind design and included blinded central independent radiographic response assessment. The study was sufficiently powered to demonstrate equivalence in the primary endpoint, and treatment groups were well-balanced with respect to baseline clinical, pathologic and demographic factors. The equivalence design is a robust method for demonstrating that a potential biosimilar is neither inferior nor superior to the originator product in a comparative clinical trial.^8,9,19^ Moreover, ORR is considered a sensitive endpoint for identifying potential product-related differences between a potential anti-cancer biosimilar and the originator,^19^ as it is a direct measure of drug activity.^20^ Sensitivity analyses for the efficacy outcomes yielded results that were consistent with the main findings. The finding of similar peak and trough levels of PF-05280014 and trastuzumab-EU across multiple doses complements the PK similarity conclusion of the previous single-dose PK study in healthy male volunteers.^12^

The incidence of patients testing positive for ADAs prior to treatment initiation (8.6% in the PF-05280014 group and 4.0% in the trastuzumab-EU group) is not without precedent. In an analysis of the immunogenicity of IV and subcutaneous formulations of reference trastuzumab in the phase III HannaH study, for example, 5.9% of patients in the IV arm and 4.2% in the subcutaneous arm were positive for ADAs to trastuzumab at baseline.^21^ Indeed, pre-existing ADAs have been detected in treatment-naïve patients across several biotherapeutic modalities.^22^ Importantly, in our study, all patients tested negative for ADAs after study treatment initiation, except two patients (one from each group). This low incidence of immunogenicity during treatment in both groups is reassuring, and consistent with results from other comparative studies of PF-05280014 and reference trastuzumab, in both healthy volunteers^12^ and patients with early breast cancer.^13^ Overall, the safety findings in the current trial were consistent with those expected of reference trastuzumab,^1,2^ with no notable differences between groups.

Although the current study assessed PF-05280014 or trastuzumab-EU in combination with paclitaxel, current standard first-line treatment for HER2+ MBC involves dual HER2 blockade with the combination of trastuzumab and pertuzumab added to chemotherapy.^23,24^ Additionally, the proportion of patients with prior treatment with trastuzumab in the study (9.4% in the PF-05280014 group and 11.0% in the trastuzumab-EU group) may be lower than that expected in routine clinical practice in some countries. However, neither of these factors compromises the study’s aim of assessing the similarity between PF-05280014 and trastuzumab-EU.

The ORRs observed in the current study were within the range of those in previous randomised studies of reference trastuzumab used in combination with a taxane for first-line treatment of MBC. For example, in a randomised phase II trial, Gasparini et al^17^ reported an ORR of 75.0% (95% CI: 62.1–85.3%) in patients treated with weekly trastuzumab and weekly paclitaxel 80 mg/m^2^. In a separate randomised phase II trial, Marty et al^18^ observed an ORR of 61% (95% CI: 50–71%) in patients treated with weekly trastuzumab plus 3-weekly docetaxel 100 mg/m^2^. Additionally, in a randomised phase III trial, Slamon et al^3^ noted an ORR of 41% (95% CI: 31–51%) in patients treated with weekly trastuzumab combined with 3-weekly paclitaxel 175 mg/m^2^. Comparisons of ORRs across separate trials should be made with caution, however, because rates could be confounded by differences in patient characteristics, prior treatment patterns, and methodological aspects, including the specific dosing regimen and whether response was assessed centrally or by the investigator.

Regulatory guidance on confirmatory clinical trials of potential biosimilars recommends conducting such studies in a population that is sensitive for the detection of product-related differences between the biosimilar and originator.^8,9,19^ HER2+ MBC is a condition for which the use of trastuzumab is well-established and globally accepted. In addition to the current study, MBC has been used as the setting for comparative clinical studies of other trastuzumab biosimilars.^25,26^ One of these, MYL-1401O, was licensed by the US Food and Drug Administration in December 2017, as trastuzumab-dkst.^27^ In a randomised trial of 500 patients without previous treatment for HER2+ MBC, this biosimilar demonstrated equivalence to reference trastuzumab in ORR at 24 weeks, when both treatments were administered 3-weekly and in combination with a taxane.^25^ Equivalence was tested via both the ORR ratio and the ORR difference; the equivalence margin for the ORR ratio was 0.81–1.24, and was thus similar to the margin used in our study.^25^ Another product, BCD-022, demonstrated non-inferiority to reference trastuzumab in ORR when each treatment was given 3-weekly in combination with paclitaxel in a randomised trial of 126 patients with HER2+ MBC.^26^

PF-05280014 has also been compared with trastuzumab-EU in the neoadjuvant treatment of operable HER2+ breast cancer.^13^ The neoadjuvant study was powered to demonstrate non-inferiority of PF-05280014 to trastuzumab-EU in the proportion of patients with steady-state trough concentrations > 20 μg/ml; as secondary endpoints, pathologic complete response (pCR) rate and ORR were found to be similar between treatments.^13^ These findings in the sensitive setting of early breast cancer help in addressing concerns voiced by some commentators regarding the sensitivity and homogeneity of a population with MBC for an assessment of potential trastuzumab biosimilars.^28^ Other trastuzumab biosimilars have also been studied in the neoadjuvant setting.^29–32^ ABP 980, CT-P6 and SB3, for example, all obtained marketing authorisation in Europe based on submissions that included data from trials comparing the respective biosimilar with reference trastuzumab when each was combined with neoadjuvant chemotherapy in an early breast cancer population.^29–35^ The primary endpoint in the trials of ABP 980 and CT-P6 was total pCR rate, while the study of SB3 utilised breast pCR rate.^29,31,32^ It should be noted that regulatory authorities have not specified a preferred setting for comparative clinical trials of trastuzumab biosimilars, and sponsors are expected to provide scientific justification for the study design chosen. This likely explains the differences among the clinical trial designs adopted for trastuzumab biosimilars.

Biosimilars are expected to have an important role in improving patient access to biologics and in helping to address increasing healthcare expenditure; both issues are pressing in the management of cancer.^36–38^ In a survey of oncologists from the USA, Mexico, Turkey, Russia and Brazil, for example, it was reported that access to trastuzumab was limited by barriers related to insurance coverage, availability of supply and cost to the patient.^6^ Approximately half of respondents reported that they would increase their use of trastuzumab if a lower-cost biosimilar version were available.^6^ Indeed, evidence from Europe, where some biosimilars have been available for >10 years, has identified both increased treatment utilisation and reduced treatment-day prices following the introduction of biosimilar competition in several therapeutic classes.^39^

Comparative clinical studies are the final step in the biosimilar development process. Rather than seeking to demonstrate *de novo* efficacy and safety, such studies aim to confirm a lack of clinically meaningful differences between the biosimilar and originator following a demonstration of similarity in earlier studies, including extensive, foundational structural and functional analyses.^8,19^ In the current study, when used as first-line treatment for HER2+ MBC, PF-05280014 plus paclitaxel demonstrated equivalence to trastuzumab-EU plus paclitaxel in the primary efficacy endpoint of ORR, with no notable differences in safety or immunogenicity profile. Furthermore, no statistically significant differences in PFS, OS or DOR were observed. As part of the totality of the evidence for assessing biosimilarity, these results are consistent with, and build upon, earlier analytical, non-clinical and clinical comparisons of PF-05280014 and reference trastuzumab.^11–13^

## Electronic supplementary material

Supplementary information

## Data Availability

Upon request, and subject to certain criteria, conditions and exceptions (see https://www.pfizer.com/science/clinical-trials/trial-data-and-results for more information), Pfizer will provide access to individual de-identified participant data from Pfizer-sponsored global interventional clinical studies conducted for medicines, vaccines and medical devices (1) for indications that have been approved in the US and/or EU or (2) in programs that have been terminated (i.e., development for all indications has been discontinued). Pfizer will also consider requests for the protocol, data dictionary, and statistical analysis plan. Data may be requested from Pfizer trials 24 months after study completion. The de-identified participant data will be made available to researchers whose proposals meet the research criteria and other conditions, and for which an exception does not apply, via a secure portal. To gain access, data requestors must enter into a data access agreement with Pfizer.
