# Supplementary material for: PF-05280014 (a trastuzumab biosimilar) plus paclitaxel compared with reference trastuzumab plus paclitaxel for HER2-positive metastatic breast cancer: a randomised, double-blind study
Source: Br J Cancer. 2018 Dec 20;120(2):172–82. doi: 10.1038/s41416-018-0340-2 (PMC6342915; doi:10.1038/s41416-018-0340-2)
Supplement: Supplementary file 1 — Supplementary information [file 41416_2018_340_MOESM1_ESM.pdf]

SUPPLEMENTARY INFORMATION

PF-05280014 (a trastuzumab biosimilar) plus paclitaxel compared with reference trastuzumab plus paclitaxel for HER2-positive metastatic breast cancer: a randomised, double-blind study

Mark D. Pegram[1], Igor Bondarenko[2], Marina Moreira Costa Zorzetto[3], Sachin Hingmire[4], Hirotaka Iwase[5], Petr V. Krivorotko[6], Keun Seok Lee[7], Rubi K. Li[8], Joanna Pikiel[9], Rajesh Aggarwal[10], Reginald Ewesuedo[11]*, Amy Freyman[11], Ray Li[12], Alicia Vana[13], Donghua Yin[14], Charles Zacharchuk[11] and Elizabeth Tan-Chiu[15]

[1]Stanford Comprehensive Cancer Institute, Stanford University School of Medicine, 900 Blake Wilbur, Stanford, CA 94305, USA; [2]Oncology and Medical Radiology Department, Dnipropetrovsk Medical Academy, 31 Blyzhnya Street, Dnipro, 49102, Ukraine; [3]Department of Clinical Oncology, Barretos Cancer Hospital, Rua Antenor Duarte Villela, 1331 – Dr. Paulo Prata, Barretos – São Paulo, 14784-400, Brazil; [4]Department of Oncology, Deenanath Mangeshkar Hospital and Research Centre, Erandawne, Pune, Maharashtra 411004, India; [5]Department of Breast and Endocrine Surgery, Kumamoto University Graduate School of Medical Science, 1-1-1, Honjo, Chuo-ku, Kumamoto, 860-8556, Japan; [6]Department of Breast Tumors, Petrov Research Institute of Oncology, 68 Leningradskaya Street, Pesochny, 197758, Saint Petersburg, Russian Federation; [7]Center for Breast Cancer, National Cancer Center, 323 Ilsan-ro, Ilsandong-gu, Goyang-si, Gyeonggi-do, Republic of Korea; [8]Section of Medical Oncology, Cancer Institute, St. Luke's Medical Center, 279 E Rodriguez Sr. Ave, Quezon City, 1112 Metro Manila, Philippines; [9]Copernicus, Wojewódzkie Centrum Onkologii, Al. Zwycięstwa 31/32, 80-210, Gdańsk, Poland; [10]Pfizer Essential Health, Worldwide Safety, Pfizer Inc, 235 East 42nd Street, New York, NY 10017, USA; [11]Pfizer Essential Health, Biosimilars Clinical R&D, Pfizer Inc, 610 Main Street, Cambridge, MA 02139, USA; [12]Early Clinical Development Statistics, Pfizer Inc, 1 Portland Street, Cambridge, MA 02139, USA; [13]Pfizer Essential Health, Biosimilars Clinical R&D, Pfizer Inc, 10777 Science Center Drive, San Diego, CA 92121, USA; [14]Clinical Pharmacology, Pfizer Inc, 10777 Science Center Drive, San Diego, CA 92121, USA; [15]Medical Oncology, Florida Cancer Research Institute, 201 NW 82nd Avenue, Suite 102, Plantation, FL 33324, USA

***Correspondence:** Dr Reginald Ewesuedo, Pfizer Essential Health, Biosimilars Clinical R&D, Pfizer Inc, 610 Main Street, Cambridge, MA 02139, USA. Phone: +1 617 803 4614; Email: reginald.ewesuedo@pfizer.com

Supplementary Methods S1. Determination of HER2+ status

Determination of human epidermal growth factor receptor 2 (HER2)-positive (HER2+) status using a sponsor-approved analytical test method (see below) was required to be documented in the patient's source documentation. If HER2 status was not available, or had been determined via a test that was not sponsor-approved, eligibility was documented before randomisation either by a sponsor-provided central laboratory or the use of HER2 local testing using both an immunohistochemistry (IHC) and an *in situ* hybridisation analytical test, neither of which were considered sponsor approved. The results from both assays were required to be unequivocal (i.e., the IHC result must have been categorised as IHC3+).

Sponsor-approved HER2 assays
HER2 results based on one of the following commercial kit assays were acceptable for the purposes of study entry. Of note, additional HER2 assays approved by the US Food and Drug Administration and not listed below were also considered acceptable for the purposes of study entry.

IHC approved assay	FISH approved assay	CISH approved assay	DISH approved assay
HercepTest™ (Dako)	PathVysion HER2 DNA Probe Kit (Abbott Molecular)	SPOT-Light® HER2 CISH™ Kit (Invitrogen Corporation)	INFORM HER2 Dual ISH DNA Probe Cocktail Assay (Ventana)
Pathway™ Her2 (Ventana)	INFORM HER2/neu Probe (Ventana)	HER2 CISH PharmDx™ Kit (Dako)	
Bond™ Oracle™ HER2 IHC System (Leica Biosystems)	Dakocytomation HER2 FISH PharmDx™ Kit (Dako)		
PATHWAY HER-2/neu (Ventana)			

Abbreviations: CISH=chromogenic *in-situ* hybridisation; DISH=dual *in-situ* hybridisation; FISH=fluorescent *in-situ* hybridisation.

Central evaluation of HER2 status
For all patients, tumour tissue blocks or unstained slides were required to be collected and sent to the sponsor-provided central laboratory for standardised evaluation of HER2 status. For those patients who were determined to be HER2+ by a local laboratory, central review was performed retrospectively. The primary analysis for study outcome was based on all patients irrespective of HER2 status, as documented at the time of randomisation. Patients determined not to be HER2+ by central laboratory evaluation were excluded from the per protocol population.

Two laboratories were used in the central evaluation of HER2 status: South Bend Medical Foundation (South Bend, IN, USA) and NeoGenomics (Fort Myers, FL, USA). Samples were initially tested by FISH; if they were not positive using FISH, reflex IHC was used. The FISH ratio used by each laboratory for determining HER2-positivity is described below. An IHC result of IHC3+ was deemed HER2+ for both laboratories.

HER2 analysis by FISH	South Bend Medical Foundation (HER2/CEP 17 ratio)	NeoGenomics (HER2/CEP 17 ratio)
Positive	>2.2	≥2.0 with any average HER2 copy number, or <2.0 with an average HER2 copy number ≥6 signals/nucleus
Equivocal	1.8–2.2	<2.0 with an average HER2 copy number ≥4 and <6 signals/nucleus
Negative	<1.8	<2.0 with an average HER2 copy number <4.0 signals/nucleus

Abbreviation: CEP 17=chromosome enumeration probe 17.

Supplementary Methods S2. Inclusion and exclusion criteria

Inclusion criteria

Eligible patients were required to meet the following and all other qualifying criteria:

1. Female patients aged 18 years or older. (Where required by regulations, consent from a legally acceptable representative was required for all patients who were younger than 20 years of age.)
2. Histologically confirmed diagnosis of breast cancer.
3. Presence of metastatic disease.
4. Documentation of human epidermal growth factor receptor 2 (HER2) gene amplification or overexpression by one of the following:
   a. Gene amplification by fluorescent *in-situ* hybridisation (FISH), chromogenic *in-situ* hybridisation (CISH) or dual *in-situ* hybridisation (DISH) (as defined by the manufacturer's kit instruction); *or*
   b. Overexpression by immunohistochemistry (IHC) categorised as IHC3+; *or*
   c. Overexpression by IHC categorized as IHC2+ with FISH, CISH or DISH confirmation.

   Determination of HER2-positive status using a sponsor-approved analytical test method was required to be documented in the patient's source documentation.
   If HER2 status was unavailable or was determined using a test other than a sponsor-approved assay, eligibility was required to be documented prior to randomisation either by:
   a. The sponsor-provided central laboratory; *or*
   b. HER2 local testing using both an IHC and an *in-situ* hybridisation (ISH) analytical test, neither of which were considered sponsor approved. The results from both assays were required to be unequivocal (i.e., IHC result was required to be categorized as IHC3+).
5. Available tumour tissue (i.e., formalin-fixed paraffin-embedded blocks or unstained slides) for central review of HER2 status. Tumour tissue should be from metastatic disease or, if not obtainable, could be from the primary tumour at the time of initial or current diagnosis.
6. Documentation of oestrogen receptor (ER) status (positive or negative) based on local laboratory or sponsor-identified central laboratory.
7. At least one measurable lesion as defined by Response Evaluation Criteria in Solid Tumours (RECIST) 1.1; measurable lesions were required to be outside prior radiation fields. The following kinds of lesions were not measurable according to RECIST 1.1: ascites, pleural or pericardial effusion, osteoblastic or osteolytic bone metastases and carcinomatous lymphangitis of the lung. The site was required to forward the radiographs to the independent central review laboratory to obtain confirmation of the presence of measurable disease prior to patient randomisation.
8. Eastern Cooperative Oncology Group (ECOG) status of 0 to 2.
9. Left ventricular ejection fraction (LVEF) within institutional range of normal, measured by either 2-dimensional echocardiogram or multi-gated acquisition scan.
10. Screening laboratory values within the following limits:
    a. Absolute neutrophil count (ANC) ≥1.5 × 10^9 cells/L (1500/mm^3);
    b. Platelet count ≥100 × 10^9 cells/L (100,000/mm^3);
    c. Haemoglobin ≥9.0 g/dl (90 g/l);
    d. Serum creatinine ≤1.5 × upper limit of normal (ULN);
    e. Total bilirubin ≤1.5 × ULN (<3 ULN if Gilbert's disease);

f. Aspartate aminotransferase (AST) and/or alanine aminotransferase (ALT) ≤2.5 × ULN (≤5 × ULN if liver metastases are present).

11. Recovered (to Grade 1 or baseline) from all clinically significant adverse effects of prior therapies (excluding alopecia).
12. Patients of childbearing potential must have agreed to use two highly effective methods of contraception, throughout the study and for 12 months after the last dose of assigned treatment. A patient was of childbearing potential if, in the opinion of the investigator, she was biologically capable of having children and was sexually active.
13. Evidence of a personally signed and dated informed consent document indicating that the patient had been informed of all pertinent aspects of the study.
14. Patients who were willing and able to comply with scheduled visits, treatment plan, laboratory tests and other study procedures.

Exclusion criteria

Patients were ineligible to participate in this study if any of the following criteria were met:

1. Patients who were investigational site staff members directly involved in the conduct of the study and their family members, site staff members otherwise supervised by the investigator or patients who were Pfizer employees directly involved in the conduct of the study.
2. Relapse within 1 year of last dose of previous adjuvant (including neoadjuvant) treatment (except endocrine therapy) and within 1 year before randomisation.
3. Prior systemic therapy for metastatic disease (except endocrine therapy).
4. Prior cumulative dose of doxorubicin of >400 mg/m^2, epirubicin dose >800 mg/m^2 or the equivalent dose for other anthracyclines or derivatives (e.g., 72 mg/m^2 of mitoxantrone). If the patient had received more than one anthracycline, then the cumulative dose must not have exceeded the equivalent of 400 mg/m^2 of doxorubicin.
5. Inflammatory breast cancer.
6. Superficial disease site that could not be assessed by radiographic method as the only site of measurable disease. Note: patients were eligible if they had superficial lesions that could be measured by computed tomography scan or magnetic resonance imaging.
7. Major surgery, radiotherapy, or any investigational agents, within 4 weeks before the administration of the first dose of study treatment.
8. Concurrent administration of other anticancer therapies. Note: bisphosphonate or receptor activator for nuclear factor κ B (RANK) ligand inhibition therapy for pre-existing bone metastases or osteoporosis was allowed; however, prophylactic use to prevent bone metastasis was not permitted.
9. Active uncontrolled or symptomatic central nervous system (CNS) metastases, as evidenced by clinical symptoms, cerebral oedema and/or progressive growth. Patients with a history of CNS metastases or cord compression were eligible if they had completed definitive treatment and had not received anticonvulsants or steroids for at least 4 weeks before first dose of study treatment. Patients with newly detected asymptomatic CNS metastases must have completed definitive treatment (e.g., radiotherapy, stereotactic surgery) before being considered for study entry. Patients with a history of carcinomatous meningitis (leptomeningeal disease) were not eligible.
10. Active uncontrolled cardiac disease, including cardiomyopathy, congestive heart failure New York Heart Association functional classification of ≥3, unstable angina or myocardial infarction within 12 months before first dose of study treatment.
11. Pre-existing Grade 2 or greater motor or sensory neuropathy.

12. History of severe hypersensitivity reaction to taxanes, trastuzumab, murine proteins or excipients in their formulations.
13. Clinical contraindication to treatment with steroids preventing use as part of paclitaxel premedication.
14. Pregnant females; breastfeeding females; females of childbearing potential who were unwilling or unable to use two highly effective methods of contraception as outlined in the protocol for the duration of the study and for 12 months after last dose of study treatment.
15. Known or demonstrated viral infection as listed below. Testing to demonstrate eligibility was required only in countries where regulations mandated testing. In all other countries, testing was considered if a patient was at risk for having undiagnosed infection (for example due to history of injection drug use or due to geographic location).
    a. Seropositivity for human immunodeficiency virus (HIV);
    b. Hepatitis B and/or hepatitis C infection (as detected by positive testing for hepatitis B surface antigen [HBsAg] or hepatitis C virus antibody [HCVAb] with confirmatory testing).
16. History of another cancer diagnosis (including contralateral breast cancer) within 5 years prior to screening for this study, with the exception of adequately treated ductal carcinoma *in-situ*, cervical carcinoma *in-situ* or basal or squamous cell skin cancer.
17. Unwilling or unable to comply with the lifestyle guidelines described in the protocol.
18. Participation in other studies involving investigational drug(s) (phases I to IV) within 4 weeks before randomisation and/or during study participation. Patients participating in observational studies not involving an investigational drug(s) and/or long-term follow-up of studies involving an investigational drug(s) in which treatment was completed ≥4 weeks before randomisation were not excluded.
19. Other severe acute or chronic medical or psychiatric conditions, including recent (within the past year) or active suicidal ideation or behaviour, or laboratory abnormality that may have increased the risk associated with study participation or study treatment administration or may have interfered with the interpretation of study results and, in the judgement of the investigator, would have made the patient inappropriate for entry into this study.

The listing above represents the final eligibility criteria for the study and is reflective of Final Protocol Amendment 3, 27 September 2016.

Supplementary Figure S1. Mean serum concentration of PF-05280014 and trastuzumab-EU vs. time (PK population) – Week 53 analysis

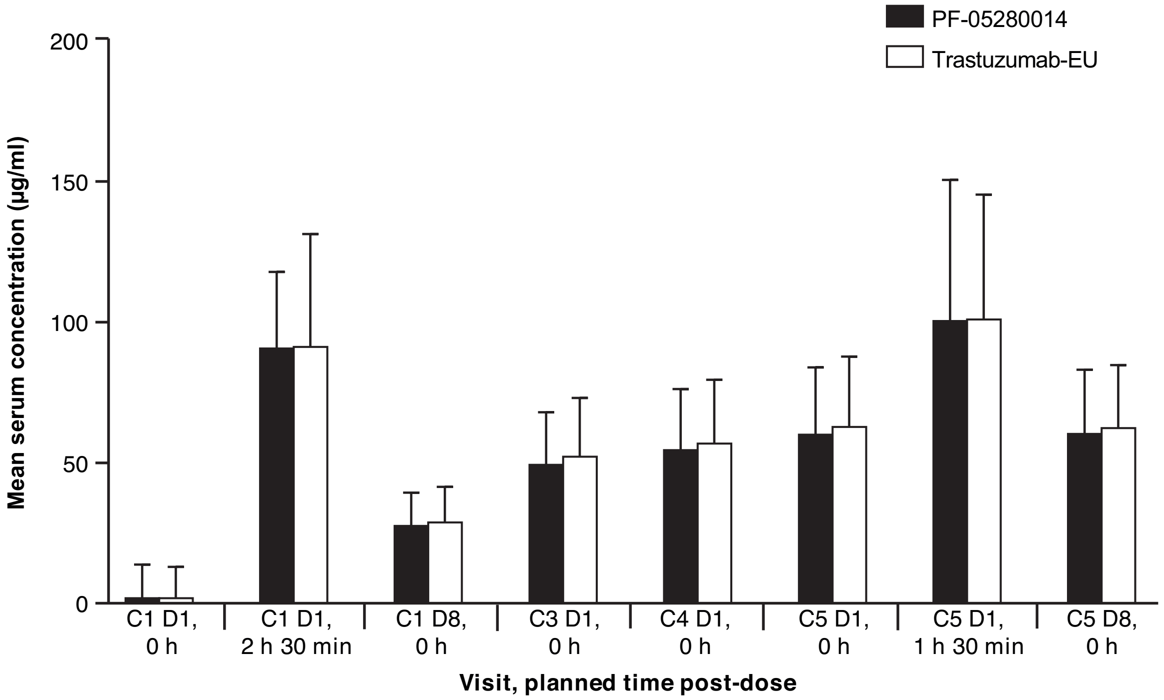

No. of observations:								
PF-05280014	349	278	339	309	304	288	204	277
Trastuzumab-EU	349	267	340	319	316	303	221	287

Error bars denote SD.

Summary statistics were calculated by setting concentration values below the lower limit of quantification (0.500 µg/ml) to zero. For the 34 patients with measurable concentrations at Cycle 1 Day 1, the range of pre-dose concentrations was 0.654–123 µg/ml; sample collection and handling procedures for these samples, prior drug treatments and other aspects were reviewed to understand the purported cause for pre-dose concentrations, and no definitive reason could be discerned.

Samples with a time deviation of >20% or any positive time deviation for the 0 h planned time point were excluded. Unplanned and end-of-treatment samples excluded.

Per protocol, patients could be on different regimens of PF-05280014 or trastuzumab-EU (either weekly or 3-weekly administration) as of Cycle 9 Day 1, but 12 patients were incorrectly switched as early as Cycle 7 Day 1; hence, concentration comparisons later than Cycle 5 could be confounded by the different regimens and are not presented.

Abbreviations: C=cycle; D=day; PK=pharmacokinetic; SD=standard deviation; trastuzumab-EU=reference trastuzumab sourced from the European Union.

Supplementary Table S1. Analysis of objective response rate derived from central radiology assessments with stratification factors (ITT population) – Week 33 analysis

	PF-05280014 plus paclitaxel (*n*=352)	Trastuzumab-EU plus paclitaxel (*n*=355)	Risk ratio estimate (95% CI)[a]
Objective response rate[b]			
n (%)	220 (62.5)	236 (66.5)	0.940
(95% CI)	(57.2–67.6)	(61.3–71.4)	(0.839–1.044)
Complete response, *n* (%)	10 (2.8)	13 (3.7)	
Partial response, *n* (%)	210 (59.7)	223 (62.8)	

The stratification factors at randomisation, prior trastuzumab exposure and oestrogen receptor status, were considered in the calculation of the CI of the risk ratio.
[a]Risk ratio and associated 95% CI were based on the Miettinen and Nurminen method.
[b]Defined as the percentage of patients within each treatment group who achieved complete response or partial response by Week 25 that was subsequently confirmed by Week 33 (or early discontinuation), in accordance with RECIST 1.1.

Abbreviations: CI=confidence interval; ITT=intent-to-treat; RECIST=Response Evaluation Criteria In Solid Tumours; trastuzumab-EU=reference trastuzumab sourced from the European Union

Supplementary Table S2. Analysis of objective response rate derived from central radiology assessments (PP population) – Week 33 analysis

	PF-05280014 plus paclitaxel (*n*=280)	Trastuzumab-EU plus paclitaxel (*n*=285)	Risk ratio estimate (95% CI)[a]
Objective response rate[b]			
n (%)	199 (71.1)	212 (74.4)	0.955
(95% CI)	(65.4–76.3)	(68.9–79.4)	(0.862–1.057)
Complete response, *n* (%)	8 (2.9)	10 (3.5)	
Partial response, *n* (%)	191 (68.2)	202 (70.9)	

[a]Risk ratio and associated 95% CI were based on the Miettinen and Nurminen method.
[b]Defined as the percentage of patients within each treatment group who achieved complete response or partial response by Week 25 that was subsequently confirmed by Week 33 (or early discontinuation), in accordance with RECIST 1.1.

Abbreviations: CI=confidence interval; PP=per protocol; RECIST=Response Evaluation Criteria In Solid Tumours; trastuzumab-EU=reference trastuzumab sourced from the European Union

Supplementary Table S3. Analysis of objective response rate derived from investigator assessments (ITT population) – Week 33 analysis

	**PF-05280014 plus paclitaxel (*n*=352)**	**Trastuzumab-EU plus paclitaxel (*n*=355)**
Objective response rate[a]		
n (%)	232 (65.9)	236 (66.5)
(95% CI)	(60.7–70.9)	(61.3–71.4)
Complete response, *n* (%)	19 (5.4)	17 (4.8)
Partial response, *n* (%)	213 (60.5)	219 (61.7)

[a]Defined as the percentage of patients within each treatment group who achieved complete response or partial response by Week 25 that was subsequently confirmed by Week 33 (or early discontinuation), in accordance with RECIST 1.1.

Abbreviations: CI=confidence interval; ITT=intent-to-treat; RECIST=Response Evaluation Criteria In Solid Tumours; trastuzumab-EU=reference trastuzumab sourced from the European Union

Supplementary Table S4. Analysis of secondary efficacy endpoints – Week 53 analysis

	PP population		ITT population	
	**PF-05280014 plus paclitaxel (*n*=280)**	**Trastuzumab-EU plus paclitaxel (*n*=285)**	**PF-05280014 plus paclitaxel (*n*=352)**	**Trastuzumab-EU plus paclitaxel (*n*=355)**
**Progression-free survival**				
Progressed or died, *n* (%)	116 (41.4)	126 (44.2)	144 (40.9)	148 (41.7)
Censored, *n* (%)	164 (58.6)	159 (55.8)	208 (59.1)	207 (58.3)
Median (95% CI),[a] months	12.42 (12.02–13.40)	12.06 (11.79–NE)	12.16 (11.93–12.48)	12.06 (11.79–NE)
HR[b] (95% CI)	0.94 (0.73–1.21)		1.00 (0.80–1.26)	
P-value[c]	0.307		0.505	
**Overall survival**				
Deaths, *n* (%)	33 (11.8)	32 (11.2)	42 (11.9)	43 (12.1)
Censored, *n* (%)	247 (88.2)	253 (88.8)	310 (88.1)	312 (87.9)
Median (95% CI),[a] months	NE	NE	NE	NE
HR[b] (95% CI)	1.079 (0.661–1.759)		1.004 (0.655–1.539)	
P-value[c]	0.619		0.507	
**Duration of response**				
Confirmed response, *n* (%)	202 (72.1)	213 (74.7)	224 (63.6)	238 (67.0)
Confirmed response status[d]				
With subsequent progression or death, *n* (%)	64 (31.7)	76 (35.7)	73 (32.6)	81 (34.0)
Without subsequent progression or death (censored), *n* (%)	138 (68.3)	137 (64.3)	151 (67.4)	157 (66.0)
Median (95% CI),[a] months	11.27 (10.41–11.27)	10.58 (10.22–NE)	11.27 (10.41–11.27)	10.58 (10.22–NE)
HR[b] (95% CI)	0.83 (0.59–1.16)		0.92 (0.67–1.27)	
P-value[c]	0.139		0.304	

Progression-free survival and duration of response derived from central radiology assessments.

[a]Kaplan–Meier estimate. 95% CI based on the Brookmeyer and Crowley method.

[b]Based on the Cox proportional hazards model stratified by prior trastuzumab exposure (Yes/No) and ER status (positive vs. negative). Assuming proportional hazards, an HR <1 indicates increased hazard rate for trastuzumab-EU plus paclitaxel; an HR >1 indicates increased hazard rate for PF-05280014 plus paclitaxel.

[c]One-sided *P*-value from the log-rank test stratified by prior trastuzumab exposure (Yes/No) and ER status (positive vs. negative).

[d]Percentage based on the number of patients with confirmed response.

Abbreviations: CI=confidence interval; ER=oestrogen receptor; HR=hazard ratio; ITT=intent-to-treat; NE=not estimable; PD=progressive disease; PP=per protocol; trastuzumab-EU=reference trastuzumab sourced from the European Union

Supplementary Table S5. Summary of cardiac function: evaluation of left ventricular ejection fraction by visit (safety population) – Week 53 analysis

Visit	PF-05280014 plus paclitaxel (*n*=349)	Trastuzumab-EU plus paclitaxel (*n*=353)
Screening		
n	348	353
Mean (SD)	65.3 (5.80)	65.3 (6.20)
Median (range)	65.0 (46.00–83.00)	65.0 (46.00–89.00)
Week 9		
n	310	319
Mean (SD)	64.3 (5.84)	64.5 (5.68)
Median (range)	64.0 (46.00–83.00)	65.0 (47.00–80.00)
Week 17		
n	295	304
Mean (SD)	64.0 (5.99)	64.4 (6.09)
Median (range)	64.0 (45.00–84.00)	64.0 (46.00–82.00)
Week 25		
n	272	276
Mean (SD)	63.6 (6.11)	64.2 (5.95)
Median (range)	63.0 (40.00–81.00)	64.0 (46.00–82.00)
Week 33		
n	232	233
Mean (SD)	64.1 (5.99)	63.9 (5.63)
Median (range)	64.0 (46.00–88.00)	64.0 (49.00–85.00)
Week 41		
n	92	91
Mean (SD)	65.2 (5.42)	63.8 (5.17)
Median (range)	66.0 (54.00–78.00)	64.0 (43.00–75.00)
Week 53		
n	59	65
Mean (SD)	63.6 (5.80)	64.1 (4.79)
Median (range)	64.0 (43.00–75.00)	64.0 (55.00–75.00)
End of treatment		
n	66	43
Mean (SD)	63.8 (6.33)	62.4 (6.93)
Median (range)	64.0 (44.00–76.00)	64.0 (36.00–75.00)

Unplanned readings have been excluded.

Abbreviations: SD=standard deviation; trastuzumab-EU=reference trastuzumab sourced from the European Union

Supplementary Table S6. Summary of serum HER2 ECD concentration (ng/ml) (PP population) – Week 53 analysis

		PF-05280014 plus paclitaxel				Trastuzumab-EU plus paclitaxel			
Visit	Planned time post-dose	*N*	NALQ	Mean	SD	*N*	NALQ	Mean	SD
C1 D1	0 h	280	280	159.83	298.292	282	282	184.42	546.620
C1 D8	0 h	273	272	134.04	267.181	274	274	148.72	483.346
	% CFB	273		–10.95	36.709	273		–11.74	27.737
C3 D1	0 h	253	253	21.23	37.997	266	266	23.82	54.026
	% CFB	253		–60.87	31.187	263		–59.46	34.288
C5 D1	0 h	238	238	17.91	47.535	250	250	13.47	19.005
	% CFB	238		–62.66	33.556	247		–59.04	36.877
C8 D1	0 h	209	209	16.52	52.662	220	220	12.98	14.508
	% CFB	209		–58.88	37.483	217		–59.13	40.000

Summary statistics calculated by setting concentration values below the lower limit of quantification to zero. EOT and unplanned samples have been excluded.

Abbreviations: % CFB=percent change from baseline (defined as Cycle 1, Day 1 0 h assessment); C=cycle; D=day; ECD=extracellular domain; EOT=end of treatment; HER2=human epidermal growth factor receptor 2; NALQ=number of observations above lower limit of quantification (2.5 ng/ml); PP=per protocol; SD=standard deviation; trastuzumab-EU=reference trastuzumab sourced from the European Union
